# Investigation and Prediction of Tensile, Flexural, and Compressive Properties of Tough PLA Material Using Definitive Screening Design

**DOI:** 10.3390/polym15204169

**Published:** 2023-10-20

**Authors:** Abdulsalam A. Al-Tamimi, Adi Pandžić, Edin Kadrić

**Affiliations:** 1 Industrial Engineering Department, College of Engineering, King Saud University, Riyadh 11421, Saudi Arabia; 2 Mechanical Engineering Faculty, University of Sarajevo, 71000 Sarajevo, Bosnia and Herzegovina; pandzic@mef.unsa.ba (A.P.); kadric@mef.unsa.ba (E.K.)

**Keywords:** Tough PLA, fused deposition modeling, FDM printing parameters, mechanical properties, tensile, flexural, compressive, maximum force, Young’s modulus, definitive screening design

## Abstract

The material extrusion fused deposition modeling (FDM) technique has become a widely used technique that enables the production of complex parts for various applications. To overcome limitations of PLA material such as low impact toughness, commercially available materials such as UltiMaker Tough PLA were produced to improve the parent PLA material that can be widely applied in many engineering applications. In this study, 3D-printed parts (test specimens) considering six different printing parameters (i.e., layer height, wall thickness, infill density, build plate temperature, printing speed, and printing temperature) are experimentally investigated to understand their impact on the mechanical properties of Tough PLA material. Three different standardized tests of tensile, flexural, and compressive properties were conducted to determine the maximum force and Young’s modulus. These six properties were used as responses in a design of experiment, definitive screening design (DSD), to build six regression models. Analysis of variance (ANOVA) is performed to evaluate the effects of each of the six printing parameters on Tough PLA mechanical properties. It is shown that all regression models are statistically significant (p<0.05) with high values of adjusted and predicted $R^{2}$. Conducted confirmation tests resulted in low relative errors between experimental and predicted data, indicating that the developed models are adequately accurate and reliable for the prediction of tensile, flexural, and compressive properties of Tough PLA material.

## 1. Introduction

Additive manufacturing (AM) technology in the past two decades has shown great and promising performance in industry and academia sectors. The advantages of this technology overcome numerous current manufacturing challenges such as expensive tooling, hard-to-manufacture complex parts, and part consolidation [1]. In general, AM technology is named, what you see is what you get (WYSIWYG), due to its ability to transfer the part’s digital copy into a real 3D physical one [2]. This is achieved by cross-sectioning the digital copy into multiple layers and depositing the material in a layer-by-layer fashion until the final build is completed. Fused deposition modeling (FDM) or filament fused fabrication (FFF) is one of the most sought-after techniques in AM due to its relatively low cost, availability of material and branched applications. FDM operates to deposit material, usually plastic, through melting, and extrusion procedures similar to how a toothpaste is squeezed out of its tube. The raw material is formed as filament and is gathered in a spool, inserted, and guided into the machine extruder head through gears and into the hot end chamber to melt the material. The melted filament is pushed in a controlled pressure into a nozzle forming small filament shape depositions. During material deposition, the extruder head is moved in high precision in the *x*-*y* axis to form a single cross-sectional layer and then the machine moves in a controlled *z*-axis movement (e.g., layer height). Such as with any manufacturing technique, FDM’s part quality, performance, and accuracy are highly dependent on several processing parameters. To name a few, layer height, printing speed, melting temperature, build platform temperature, infill density, part wall thickness, and build orientation are the most common parameters [3]. These printing parameters are usually determined by the type of material and machine capabilities. The layer height controls the number of how many digital models will be cross-sectioned and the value of the *z*-axis movement and usually ranges between 0.1 and 0.4 mm with an increment of 0.05 mm. Previous studies have shown that larger heights produce negative outcomes [4]. Infill density determines the amount of material used inside the part where 100% infill means that the part is fully dense (i.e., solid) and occupies the inside. This parameter is important in FDM as not all functional parts are required to be manufactured as complete solid allowing to reduce cost by using less material without compromising the part functionality [5].

The FDM process was mainly developed to print thermoplastic materials such as polyamide (PA), acrylonitrile butadiene styrene (ABS), poly-lactic acid (PLA) polycarbonate (PC), and polyethylene terephthalate glycol (PETG). The advances in the FDM process capability allow us to develop and print metallic materials and novel materials of composites, multi-material, and functional graded material [6,7,8]. PLA and ABS are the two most utilized parent materials in FDM due to their low cost, printability, convenient functionality, and mechanical performance. PLA has become an essential parent material for every FDM consumer and is supplied by the manufacturers of FDM as a trial-based material. This is mainly due to having better printability than ABS without requiring an enclosed machine envelope to preserve its shape during printing without warping or shrinking. PLA material has played a vital role in many different applications, due to its biodegradability [9], such as medical implants [10], fibers (e.g., clothing) [11], and food packaging [12,13]. However, natural PLA is brittle with a lower thermal expansion coefficient. Commercially, this led to the further development of PLA material with newer versions such as PLA+ from eSUN and Tough PLA from UltiMaker aiming to improve the parent’s material, impact resistance, toughness, and layer adherence to expand the suitability for various engineering applications [14,15,16]. Tough PLA mechanical properties were investigated in terms of its tensile properties considering infill structure [17], raster angles and infill speeds [16]. In other cases, Tough PLA was used for automobile power transmission systems and examined its tensile and flexural properties with the manufacturer’s printing parameters [18]. Tough PLA was also studied for low-cost prosthetic sockets and its tensile properties were evaluated in annealing conditions [19]. However, the effect of different printing parameters on Tough PLA’s tensile, flexural, and compressive properties was not investigated and is worth exploring to understand the commercially available material.

One of the most important properties of the 3D-printed final products is the mechanical strength which ensures proper functionality [3]. As the 3D-printed part’s mechanical performance is significantly dependent on printing parameters, several studies have considered its range of effects using design of experiment (DoE) approaches due to its ability to accurately measure the influence of each printing parameter statistically [3]. DoE allows simultaneous variation of all considered experimental parameters to obtain sufficient information by performing a minimal number of experiments [20]. The traditional approach in DoE includes performing screening experiments to determine significant main effects, followed by full factorial or response surface methodology experiments to optimize considered responses. Auffray et al. [21] have used the Taguchi DoE approach to study the influence of infill pattern, layer height, infill density, printing velocity, raster orientation, outline overlap, extruder temperature, and the interactions of infill pattern + layer height, infill pattern + infill density, and layer height + infill density on PLA part’s tensile Young’s modulus and yield strength. The study has shown that the infill density, infill pattern, printing velocity, and printing orientation significantly affect the tensile properties of PLA 3D printed parts. Kechagias and Vidakis [22] investigated the efficiency of Box–Behnken design (BBD) and full factorial design (FFD) approaches in 3D printing of PA12’s ultimate tensile strength considering raster angle, layer thickness, and nozzle temperature with three levels each. The authors presented that both approaches are adequate for parameter analysis with mean absolute error percentage (MAPE) of 5.3% for BBD and 5.2% for FFD and root-mean-square error (RMSE) of 2.75 for BBD and 2.44 for FFD.

However, traditional DoE methods are resource and time-expensive, especially in experiments with a relatively large number of factors. To overcome the disadvantages of the conventional approaches, an alternative DoE method named definitive screening design (DSD) was introduced [23]. Using DSD, the screening of factors and optimization of responses are performed in one step, i.e., one experiment. This is achieved by the convenient properties of DSD which allow the estimation of main effects, two-factor interactions, and quadratic effects, simultaneously. All main effects are orthogonal to all quadratic and all two-factor interaction effects; however, though correlated, quadratic effects are not completely confounded with two-factor interaction effects. DSD allows variation of continuous factors on three (low, middle, and high) and categorical on two levels (low and high). In a review of the literature, DSD has been conducted in FDM to determine the impact of layer height, deposition angle, infill, extrusion speed, extrusion temperature, air gap, build orientation, road width, number of contours, and bed temperature for PLA and PC-ABS on creep deformation, flexural properties, tribological properties, surface roughness, and dimensional accuracy [24,25,26,27,28,29]. In addition, DSD was used to develop regression prediction models to predict the properties of FDM material and presented with adequate predictability [30,31]. However, DSD has yet to be employed to investigate and predict the printing parameters’ impact on Tough PLA. Definitive screening design was used as the main experimental design for this study due to its advantages of requiring a minimal number of experimental runs, leading to efficient utilization of resources and time, as well as its capability to perform effects screening and optimization of responses in one-step experimental design.

The aim of this study is to investigate the impact of six different FDM printing parameters, particularly, layer height, wall thickness, infill density, build plate temperature, printing speed, and printing temperature on tensile, flexural, and compressive properties of Tough PLA material using definitive screening design methodology. Employing DSD methodology, the authors have also developed sufficiently accurate models for the prediction of Tough PLA mechanical properties using regression analysis. The remainder of this paper is organized as follows. Section 2 presents the used methodology, including specimen designs and printing parameters selection, mechanical properties testing procedure, experimental design using DSD, analysis of variance, and confirmation tests used to validate developed models. In Section 3, the main results of the study are presented with a detailed discussion of the effects of the six printing parameters on the mechanical properties of the Tough PLA material. Finally, in Section 4, the main conclusions of this study are presented.

## 2. Materials and Methods 

In this study Tough PLA material, by UltiMaker (UltiMaker, Utrecht, The Netherlands), was investigated. Tough PLA is also known as a technical PLA material that has a similar toughness to ABS. It has good printability as PLA material and good mechanical properties that can be used for printing functional prototypes, tooling, manufacturing aids, and technical products at large sizes. Also, it has a similar impact strength to ABS and is less brittle than regular PLA. Mechanical properties of FDM printed Tough PLA material are presented in Table 1.

**Figure 1:**
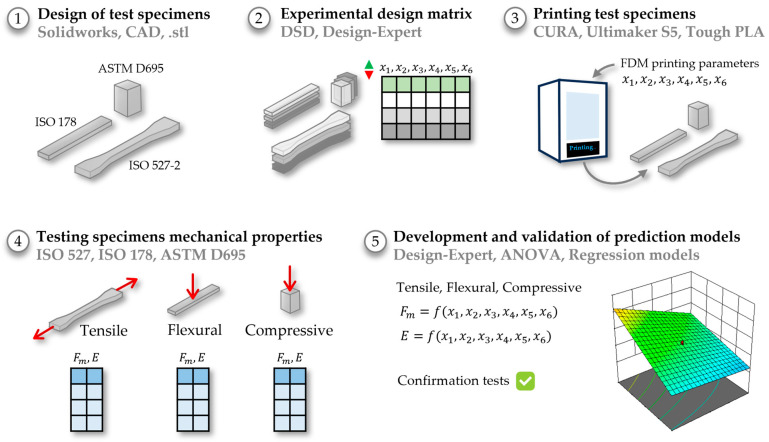
Experimental methodology.

The experimental procedure encompassed multiple distinct phases, and the comprehensive methodology is visually presented in Figure 1.

### 2.1. Specimen’s Design and Printing

This study investigates the effects of six FDM printing parameters (factors), namely layer height, wall thickness, infill density, build plate temperature, printing speed and printing temperature on the maximum force ($F_{m}$), and Young’s modulus (E) of the tensile ($F_{m,t}$, $E_{t}$), flexural ($F_{m,f}$, $E_{f}$), and compressive ($F_{m,c}$, $E_{c}$) properties, selected as FDM process responses. The selected factors have been shown to be significantly important in 3D printing applications [36]. Table 2 shows the six FDM printing parameters and the range of their values (levels).

**Figure 2:**
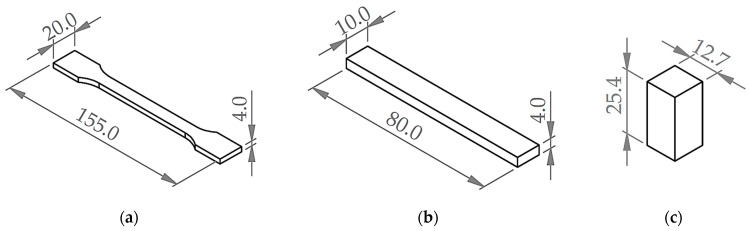
Design of specimens used for testing of (**a**) tensile properties, designed according to ISO 527-2; (**b**) flexural properties, designed according to ISO 178; and (**c**) compressive properties, designed according to ASTM D695 (all dimensions in mm).

Each experimental run, as described in Section 2.3, represents FDM printing parameter settings used to print test specimens. Test specimen’s 3D CAD models are designed in Solidworks 2023 (Dassault Systèmes, Vélizy-Villacoublay, France) according to ISO 527-2 for tensile [37], ISO 178 for flexural, and ASTM D695 [38] for compression standards as shown in Figure 2. The CAD models for each specimen were exported to .stl format and then imported in UltiMaker Cura 5.4.0 slicer (UltiMaker, Utrecht, The Netherlands) to generate G-code with printing parameters and then transferred to the UltiMaker S5 printer (UltiMaker, Utrecht, The Netherlands) for 3D printing. In order to realize the factor’s effect, the specimen’s material was fixed using Tough PLA material (Tough PLA for S series; obtained from UltiMaker, Utrecht, the Netherlands) which is considered as a material for functional prototype and tooling parts [17]. All specimens were 3D printed with predefined parameters using Cura “normal” profile settings. Specimens were printed in flat (xy) orientation with a nozzle diameter of 0.4 mm and a grid infill pattern. Specimens were printed ‘one at a time’ and were conditioned at room temperature for at least 24 h before measurement.

### 2.2. Testing Tensile, Flexural, and Compressive Properties of 3D-Printed Specimens

Tensile, flexural, and compression testing of specimens was performed according to ISO 527, ISO 178, and ASTM D695, respectively, using Shimadzu AGS-X 10 kN universal testing machine (Shimadzu Corporation, Kyoto, Japan). According to the standards and preliminary experiments, the testing speed was set at 5 mm/min. The maximum force ($F_{m}$, N) and Young’s modulus (E, GPa) data were measured and recorded in Shimadzu Trapezium-X software version 1.5.2 (Shimadzu Corporation, Kyoto, Japan), and then transferred to Design-Expert software version 13 (Stat-Ease, Inc., Minneapolis, MN, USA) for assessment of FDM printing parameters impact.

### 2.3. Experimental Design Using Definitive Screening Design

An experimental matrix for DSD can be constructed using the algorithm proposed by Jones and Nactsheim [23]. However, for the number of factors greater than 10, the conference matrices approach provides better D-efficiency of design [39]. The number of required experimental runs in DSD depends on the number and type of factors. Thus, for m even and continuous factors, the required number of experimental runs is 2m+1, and for m odd, it is 2m+3. All runs have one factor at its middle level, while others are at their extreme levels (low or high), except the central point run where all factors are at their middle levels. In experiments with mixed factor types (continuous and categorical), two additional runs are required in which all continuous factors are set on their middle levels and categorical on their extreme levels (low and high). If m is even, then the required number of runs is 2m+2, and when m is odd, then 2m+4 runs.

Considering the effects of six continuous factors described in Table 1, the minimum required number of experimental runs is 13, including one central point. In this study, the original experimental matrix is augmented with four additional central points (experimental runs 14–17 in Table 3) to increase degrees of freedom and to provide a better estimation of lack of fit. The augmented DSD experimental matrix, with measured values of corresponding responses, is shown in Table 3. Design of the experimental matrix and the statistical analysis of the experimental data are performed using Design-Expert software version 13 (Stat-Ease, Inc., Minneapolis, MN, USA). Design-Expert is specialized statistical software widely used for the design of experiments, with an intuitive interface, powerful and robust statistical tools, and feature-rich graphics. Utilizing Design-Expert enables factor screening, modeling, and optimization of responses using well-established methods such as factorial, response surface, and mixture designs.

### 2.4. Analysis of Variance

To analyze the experimental data and develop regression models for all six responses Design-Expert software version 13 (Stat-Ease, Inc., Minneapolis, MN, USA) was used. Analysis of variance (ANOVA) is used to assess the statistical significance of models and factors at 0.05 significance level. The statistical significance of models and factors is assessed using *F*-values and their corresponding *p*-values. Models and factors with *p*-values less than 0.05 (95% confidence interval) have been considered statistically significant. Visual representations of printing parameters’ effect on mechanical properties are analyzed in main effects (perturbation) plots. These plots provide clear and quick visual identification of each factor’s effect on the response and enable identification of the most influential factors. They show how increasing each factor, from level −1 to +1, affects the response. The difference between the response value at factor level +1 and −1 represents the effect of the factor, where a larger difference represents a larger factor effect.

The least-squares methodology has been used to fit the experimental data to a quadratic regression model, given by:

(1) $$y=\beta_{0}+\sum \beta_{i}x_{i}+\sum \beta_{ii}x_{i}^{2}+\sum \sum \beta_{ij}x_{i}x_{j}+\varepsilon$$

where y is considered response, $x_{i}$ and $x_{j}$ are factors, $\beta_{0}$ is intercept, $\beta_{i}$, $\beta_{ii}$, and $\beta_{ij}$ are coefficients of linear, quadratic, and two-factor interaction terms, respectively, and ε is a random normally distributed error. 

The model terms were determined considering corrected Akaike information criterion (AICc) and manual (i.e., main effects) approaches, whichever was suitable to provide adequate and accurate models [40]. Only statistically significant terms are included in the models (p<0.05), while insignificant ones (p>0.05) are omitted, such that models’ hierarchy and heredity are preserved. This approach should provide optimized models with satisfactory performances. Models’ performances are assessed using adjusted $R_{adj}^{2}$ and predicted $R_{pred}^{2}$ coefficients of determination, RMSE, and adequate precision. Predicted and experimental values are represented by different colors where blue colors represent the lowest value of the response and red colors the highest.

### 2.5. Confirmation Tests

To verify each model’s adequacy for the prediction of respective responses, confirmation tests are performed on three specimens printed with randomly selected values of FDM printing parameters. For assessment of the disagreement between experimental and predicted values, the percentage relative error (RE) is used and calculated as follows:

(2) $$RE=\frac{\left|y-\hat{y}\right|}{y}\cdot 100\%$$

where y and $\hat{y}$ are the experimental and predicted values of the corresponding response, respectively.

## 3. Results and Discussion

### 3.1. ANOVA and Regression Models for Prediction of Tough PLA Mechanical Properties

Analysis of variance (ANOVA) is performed for all six models and results are shown in Table 4, Table 5, Table 6, Table 7, Table 8 and Table 9. The results show that all models are statistically significant (p<0.05). The lack of fits for all models is not statistically significant (p>0.05) indicating an adequate selection of models. 

Regression models, with parameter values in coded units, for prediction of the tensile, flexural, and compressive maximum force $F_{m}$ and Young’s modulus E, are developed using the least squares method and are mathematically described by Equations (3)–(8). Only statistically significant terms are included in the models (p<0.05) such that the models’ hierarchy is preserved. This approach provides reduced (optimized) models and sufficient degrees of freedom for reliable estimation of lack of fit and pure errors. It can be noticed that all models include all the main effects, some of the two-factor interaction, and quadratic effects.

(3) $$F_{m,t}=1140.91-58.28x_{1}+89.78x_{2}+61.90x_{3}+38.38x_{4}-60.93x_{5}+87.12x_{6}\;-34.96x_{1}x_{2}+29.20x_{6}^{2}$$

(4) $$E_{t}=1.79-0.07x_{1}+0.07x_{2}+0.13x_{3}+0.03x_{4}-0.08x_{5}+0.10x_{6}+0.07x_{5}^{2}$$

(5) $$F_{m,f}=96.85-3.46x_{1}+4.62x_{2}+3.58x_{3}+2.71x_{4}-4.93x_{5}+5.72x_{6}-3.38x_{1}x_{2}\;-2.25x_{2}x_{3}$$

(6) $$E_{f}=2.47-0.1046x_{1}+0.0498x_{2}+0.0471x_{3}+0.0501x_{4}-0.0850x_{5}+0.1256x_{6}\;-0.0716x_{4}x_{5}+0.1007x_{4}^{2}$$

(7) $$F_{m,c}=6136.25-352.10x_{1}+536.21x_{2}+1585.67x_{3}+87.31x_{4}-365.91x_{5}\;+489.93x_{6}-276.69x_{4}x_{6}+687.07x_{5}^{2}-434.97x_{6}^{2}$$

(8) $$E_{c}=0.6266-0.0187x_{1}+0.0220x_{2}+0.0331x_{3}-0.0344x_{4}-0.0946x_{5}+0.0431x_{6}\;+0.0303x_{1}x_{3}-0.0358x_{3}x_{5}$$

**Figure 3:**
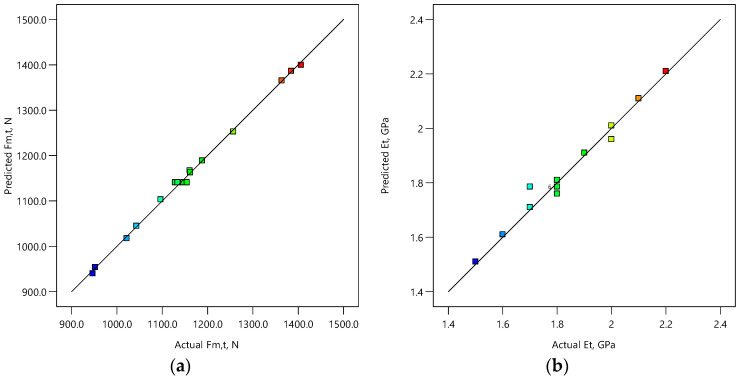

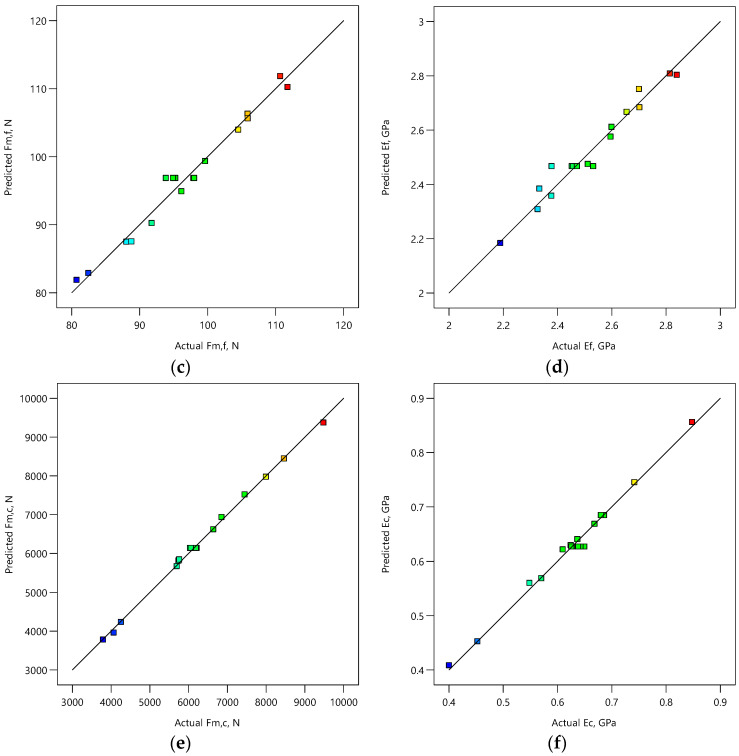
Predicted vs. actual plots for tensile (**a**) maximum force $F_{m,t}$ and (**b**) Young’s modulus $E_{t}$; flexural (**c**) maximum force $F_{m,f}$ and (**d**) Young’s modulus $E_{f}$; and compressive (**e**) maximum force $F_{m,c}$ and (**f**) Young’s modulus $E_{c}$. Color code indicates a range of low (blue) to high (red) values of the response.

Figure 3 shows the plots of the predicted vs. actual values for all considered mechanical properties. Values of mechanical properties are represented by colors in the range of blue, representing the lowest values, to red representing the highest values. The actual vs. predicted plots of the mechanical properties show high agreement and correlation between experimental and predicted data. However, it can be observed that agreement between predicted and experimental data is slightly better for models for the prediction of maximum force than for models for the prediction of Young’s modulus. This indicates that developed models are adequate for prediction of tensile, flexural, and compressive maximum force $F_{m}$ and Young’s modulus E.

The performance metrics for all six models are summarized in Table 10. Coefficients of determination $R_{adj}^{2}$ and $R_{pred}^{2}$ are in good agreement and with the difference between them of less than 0.2 (20%), which indicates that the models are expected to predict new unseen data with satisfactory accuracy [28]. Adequate precision (signal-to-noise ratio) for all models is greater than 4; hence, all models can be used to navigate design space.

### 3.2. Analysis of Effects of FDM Printing Parameters on Mechanical Properties of Tough PLA

#### 3.2.1. Tensile Properties 

**Figure 4:**
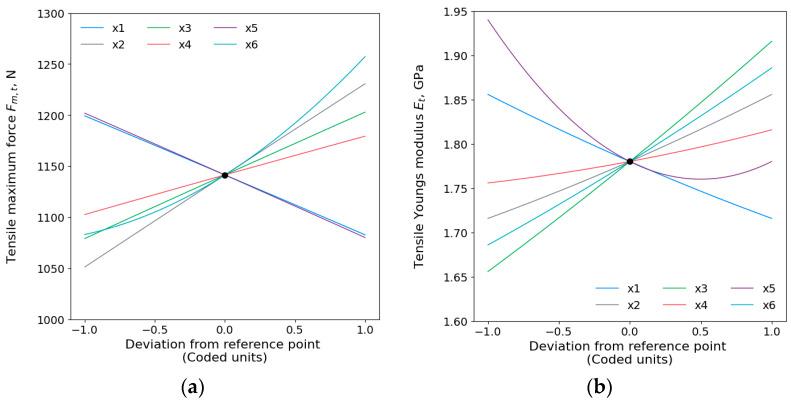
Main effects plots for tensile (**a**) maximum force $F_{m,t}$ and (**b**) Young’s modulus $E_{t}$.

Main effect plots for the tensile maximum force $F_{m,t}$ and tensile Young’s modulus $E_{t}$ are displayed in Figure 4. The order for the tensile $F_{m,t}$ in terms of the printing parameters has shown that the wall thickness ($x_{2}$) is the dominant parameter, followed by printing temperature ($x_{6}$), infill density ($x_{3}$), printing speed ($x_{5}$), layer height ($x_{1}$), build plate temperature ($x_{5}$), the interaction of layer height and wall thickness ($x_{1}x_{2}$), and the least effective parameter is the square of the printing temperature ($x_{6}^{2}$). The analysis shows that the infill density ($x_{3}$) had the highest impact on the tensile Young’s modulus $E_{t}$, then ordered by the printing temperature, printing speed, layer height, wall thickness, square of printing speed ($x_{5}^{2}$), and, lastly, by the build plate temperature.

Results present that the wall thickness has the largest effect on the tensile maximum force. The wall (shell) thickness controls the distance between the outer layer and the inner layer (i.e., the edge of the part). The increase in wall thickness presented an increase in the tensile properties. This is due to a higher wall thickness which will create a stronger part and minimize the risk of print leakage. Similar results were shown by Sukindar et al. [41], who reported that increasing the wall thickness has the dominant effect on the tensile strength of the FDM prints. On the other hand, infill density has a dominant effect on the tensile Young’s modulus. This was expected as denser parts would result in improved mechanical properties [42,43]. Higher infill rates also enhance bonding between material layers and raster while increasing printed polymer material chain resistance.

In both responses of the tensile properties, results showed that the printing temperature was the second influential parameter. The mechanical properties increased with a higher printing temperature corroborating with the literature [44]. These results are also confirmed in the findings of Gordelier et al. [45] where they concluded that higher printing temperatures provide better inter-raster and inter-layer bonding and improve the tensile strength. Although statistically significant (p<0.05), the least parameter to affect the tensile maximum force was the square of printing temperature, while build plate temperature had the least impact on the tensile Young’s modulus.

Additionally, a higher build plate temperature was found to slightly improve the mechanical properties. As the build temperatures increase, the energy is increased promoting better filament and interlayer interaction, thereby improving adhesion, and reducing internal porosity [46]. In the case of the layer height, thinner layers enhance the bonding strength and can limit the movement of nearby polymer chains [42,47,48]. In this study, the printing speeds ranged from 30 mm/s to 70 mm/s and presented that slower speed increases the mechanical properties. This was more explicit for the maximum tensile force than for Young’s modulus. The reason for this is that reduced speeds decrease the deposition time and cause more interaction and inter-layer connection between the deposited paths [16]. These findings were also confirmed for different materials such as polyetheretherketone (PEEK), where to increase the tensile strength of the printed parts, printing should be set at lower speeds [46].

#### 3.2.2. Flexural Properties

**Figure 5:**
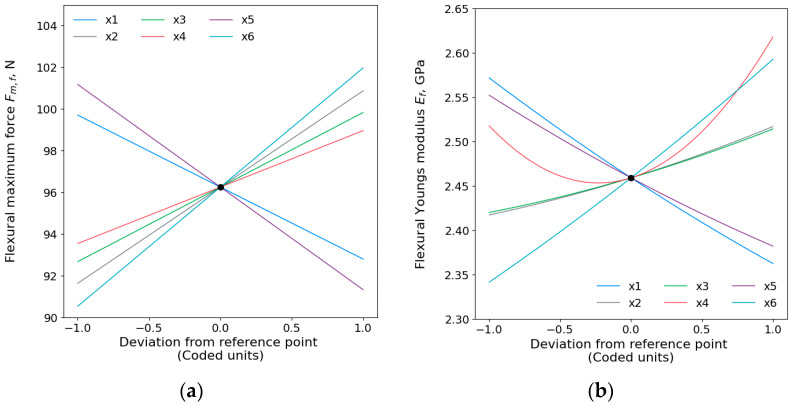
Main effects plots for flexural (**a**) maximum force $F_{m,f}$ and (**b**) Young’s modulus $E_{f}$.

Main effect plots of flexural maximum force $F_{m,f}$ and flexural Young’s modulus $E_{f}$ are shown in Figure 5. The order of printing parameters affects flexural $F_{m,f}$ where the printing temperature ($x_{6}$) has shown the dominant influence, followed by the printing speed ($x_{5}$), wall thickness ($x_{2}$), infill density ($x_{3}$), layer height ($x_{1}$), the interaction of layer height and wall thickness ($x_{1}x_{2}$), build plate temperature ($x_{4}$), and, lastly, the interaction of wall thickness and infill density ($x_{2}x_{3}$). The Young’s modulus $E_{f}$ effect order for each parameter has been obtained and presented that the printing temperature was the most influential, and then the layer height, printing speed, square of build plate temperature ($x_{4}^{2}$), the interaction of build plate temperature and printing speed ($x_{4}x_{5}$), build plate temperature, wall thickness, and infill density.

Similar trends were observed in the effect of the printing parameters on both the tensile and flexural maximum force. The printing temperature ($x_{6}$) has been shown to be the dominant on flexural maximum force and flexural Young’s modulus. At higher temperatures, the polymer would exhibit low viscosity and high fluidity improving the polymer chains to fuse together [4]. These phenomena implicate the flexural properties leading the part to withstand bending forces when printed at a higher temperature of 230 °C. The printing speed has the second highest impact on the maximum flexural force, where greater maximum force is achieved at lower speeds. A study by Christiyan et al. [47] stated that low printing speed gives better bonding with the previous layer leading to better flexural strength. For the flexural Young’s modulus, layer height was presented to have the second highest impact where smaller layer heights promote tighter interlamination, attributed to nozzle pressure [46]. The lowest effect on the flexural maximum force was observed for the interaction of wall thickness and infill density, whereas only the infill density had the least impact on Young’s modulus. However, both wall thickness and infill density had statistically significant (p<0.05) impacts on the flexural properties and were in agreement with the literature [49,50]. Increasing the build plate temperature from 30 to 90 °C resulted in an increase in flexural $F_{m,f}$ and $E_{f}$ as concluded in other studies [51,52,53].

#### 3.2.3. Compressive Properties

**Figure 6:**
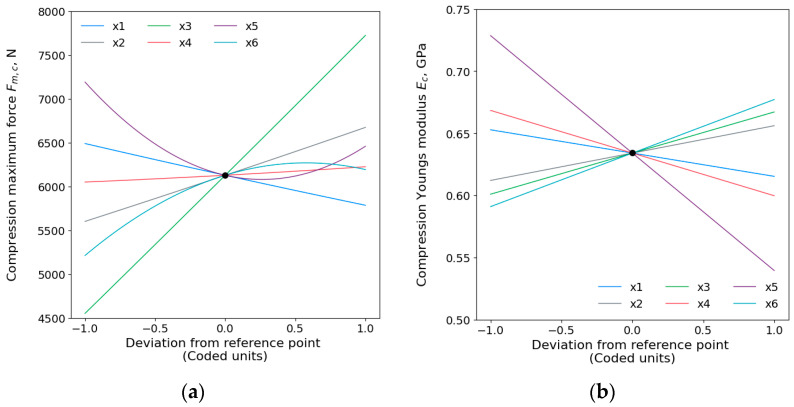
Main effects plots for compressive (**a**) maximum force $F_{m,c}$ and (**b**) Young’s modulus $E_{c}$.

Main effect plots of compressive maximum force $F_{m,c}$ and compressive Young’s modulus $E_{c}$ are depicted in Figure 6. The order of effects on compressive $F_{m,c}$ evidently shows that infill density ($x_{3}$) has the most significant influence, followed by wall thickness ($x_{2}$), printing temperature ($x_{6}$), printing speed ($x_{5}$), layer height ($x_{1}$), the square of printing speed ($x_{5}^{2}$), the square of the printing temperature ($x_{6}^{2}$), the interaction of the build plate and printing temperatures ($x_{4}x_{6}$), and the least important was the build plate temperature ($x_{6}$). The compressive Young’s modulus $E_{c}$ was affected by the considered printing parameters in the following order: printing speed, printing temperature, build plate temperature, infill density, interaction of infill density and printing speed ($x_{3}x_{5}$), the interaction of layer height and infill density ($x_{1}x_{3}$), wall thickness, and, finally, the layer height.

An increase in compressive maximum force $F_{m,c}$ and modulus $E_{c}$ can be observed when decreasing layer height and increasing wall thickness and infill density, which are in line with other studies [54,55]. This can be explained by the fact that reducing layer thickness minimizes space between material paths, increasing contact with adjacent layers, and reinforcing bonding between layers. Also, maximizing wall thickness and infill density improves compressive strength due to the rise in the material mass in each unit volume, boosting resistance. According to Petousis et al. [56], compressive strength is increased by higher build plate temperature without statistical significance. In this study, the build plate temperature showed a statistical significance (p<0.05) increasing compressive Fm, however, in a very low increase in magnitude.

With the increase in printing speed, the compressive $F_{m,c}$ and $E_{c}$ decrease. Those results are also confirmed by Yu et al.’s study [57], where the authors observed that higher printing speeds generally result in lower compressive mechanical properties. Increased speed reduces extruder uniformity and can cause instant wire breakage, leading to sample defects. Also, incomplete cooling of the upper layer during printing creates uneven bonding with the preceded layer, further reducing the material mechanical properties. Additionally, the results demonstrated an increase in compressive $F_{m,c}$ and $E_{c}$ by increasing printing temperature, which is also proven in research paper [51]. Hsueh et al. [58] confirmed that as the printing temperature increases, the compressive mechanical properties of the PLA and PETG materials increase.

#### 3.2.4. Summary of the Printing Parameters Influence on the Mechanical Properties

In Table 11 we present a summary of the printing parameters’ influence on the mechanical properties. An increase in the parameter results in an increase “+” of the response value or a decrease “−”.

### 3.3. Confirmation Tests

Confirmation test results are shown in Table 12. The lowest average percentage of relative errors is obtained for tensile properties with 3.41% and 1.10% for $F_{m,t}$ and $E_{t}$, respectively. For all the conducted mechanical tests, the obtained percentage relative errors are in the range from 3.41 to 4.51% for prediction of maximum force, and in the range from 1.10 to 7.00% for prediction of Young’s modulus. Similar to other studies using PLA, regression models developed in this study have shown satisfactory results in confirmation tests with RE less than 10%. Therefore, developed regression models can be considered adequate for the prediction of tensile, flexural, and compressive properties of 3D-printed Tough PLA material [27,28].

## 4. Conclusions

In this study, the influence of FDM printing parameters on the tensile, flexural, and compressive properties of FDM printed Tough PLA material is investigated. Definitive screening design is used for experimental design. Regression analysis is performed to develop regression models for the prediction of maximum force and Young’s modulus for all six responses, while analysis of variance is performed to determine the most influential FDM printing parameters on all six responses.

Coefficients of determination of the models for the prediction of the tensile, flexural, and compressive maximum force and Young’s modulus are high, indicating high agreement of experimental and model-predicted data. Conducted confirmation tests resulted in a low percentage of relative errors between the predicted and experimental data, validating the adequacy of the developed models.

Based on the analysis of variance, it is determined that the wall thickness is the most influential parameter on the tensile maximum force. The infill density parameter has the highest impact on the tensile Young’s modulus and the compressive maximum force. The printing temperature is the most influential parameter on the flexural maximum force and the flexural and compressive Young’s modulus.

According to the obtained performance metrics, it can be concluded that developed models are adequate for accurate and reliable prediction of maximum force and Young’s modulus for tensile, flexural, and compressive properties. It is also demonstrated that a definitive screening design is a very effective and promising experimental design method for the investigation of FDM 3D-printed materials. 

The conducted research provides a better understanding of the influence of the printing parameters on the three most significant mechanical properties of Tough PLA material. Moreover, this paper supports other researchers in the investigation of the printability of different materials to obtain improved mechanical properties.

## Figures and Tables

**Table 1:** Mechanical properties of FDM printed Tough PLA material by manufacturer [14,32,33,34,35].

Mechanical Properties	Test Method	Typical Value in xy (Flat Orientation)
Tensile (Youngs) modulus	ASTM D3039 (1 mm/min)	2797 ± 151 MPa
Tensile stress at yield	ASTM D3039 (5 mm/min)	45.3 ± 2.0 MPa
Tensile stress at break	ASTM D3039 (5 mm/min)	27.5 ± 7.8 MPa
Elongation at break	ASTM D3039 (5 mm/min)	9.4 ± 1.9%
Flexural modulus	ISO 178 (1 mm/min)	2882 ± 61 MPa
Flexural strength	ISO 178 (5 mm/min)	91.6 ± 1.3 MPa
Flexural strain at break	ISO 178 (5 mm/min)	No break (>10%)
Charpy impact strength (at 23 °C)	ISO 179-1/1eB (notched)	8.9 ± 0.8 kj/m^2^
Hardness	ISO 7619-1 (Durometer, Shore D)	80 Shore D

**Table 2:** FDM printing parameters (factors) with value ranges.

FDM Printing Parameter	Symbol	Units of Measure	Level		
			−1	0	1
Layer height	$x_{1}$	mm	0.1	0.15	0.2
Wall thickness	$x_{2}$	mm	0.8	1.2	1.6
Infill density	$x_{3}$	%	20	50	80
Build plate temperature	$x_{4}$	°C	30	60	90
Printing speed	$x_{5}$	mm/s	30	50	70
Printing temperature	$x_{6}$	°C	200	215	230

**Table 3:** DSD experimental matrix with measured responses (coded units).

Exp. Run	Factors						Tensile		Flexural		Compressive	
	$x_{1}$	$x_{2}$	$x_{3}$	$x_{4}$	$x_{5}$	$x_{6}$	$F_{m,t}$, N	$E_{t}$, GPa	$F_{m,f}$, N	$E_{f}$, GPa	$F_{m,c}$, N	$E_{c}$, GPa
1	0	1	1	1	1	1	1384.60	2.10	105.90	2.70	8461.43	0.55
2	0	−1	−1	−1	−1	−1	952.10	1.60	82.48	2.33	3795.01	0.61
3	1	0	−1	1	1	−1	946.30	1.50	80.73	2.19	4069.74	0.40
4	−1	0	1	−1	−1	1	1406.00	2.20	110.69	2.81	9482.35	0.85
5	1	−1	0	−1	1	1	1043.20	1.70	91.78	2.51	5743.38	0.57
6	−1	1	0	1	−1	−1	1363.70	2.00	111.78	2.84	7446.60	0.69
7	1	1	−1	0	−1	1	1256.90	1.90	104.53	2.60	5760.76	0.67
8	−1	−1	1	0	1	−1	1021.60	1.80	88.06	2.38	6852.19	0.45
9	1	1	1	−1	0	−1	1096.30	1.80	88.80	2.33	6641.50	0.68
10	−1	−1	−1	1	0	1	1160.50	1.80	96.17	2.70	4256.43	0.62
11	1	−1	1	1	−1	0	1188.00	2.00	105.91	2.66	7998.28	0.74
12	−1	1	−1	−1	1	0	1161.70	1.80	99.63	2.60	5697.12	0.64
13	0	0	0	0	0	0	1128.40	1.80	93.87	2.45	6212.71	0.65
14 ^1^	0	0	0	0	0	0	1142.90	1.80	97.92	2.46	6127.19	0.63
15 ^1^	0	0	0	0	0	0	1133.80	1.80	98.03	2.53	6192.94	0.64
16 ^1^	0	0	0	0	0	0	1147.20	1.80	95.27	2.47	6037.87	0.63
17 ^1^	0	0	0	0	0	0	1154.30	1.70	94.93	2.38	6061.77	0.64

^1^ Additional central point experimental runs.

**Table 4:** ANOVA for the model for prediction of tensile maximum force $F_{m,t}$.

Source	Sum of Squares	Degrees of Freedom	Mean Square	F-Value	*p*-Value	Comment
Model	2.898 × 10^5^	8	36,230.84	452.11	<0.0001	significant
Lack of Fit	212.07	4	53.02	0.49	0.7441	not sign.
Pure Error	429.03	4	107.26			
Cor Total	2.905 × 10^5^	16				

**Table 5:** ANOVA for the model for prediction of tensile Young’s modulus $E_{t}$.

Source	Sum of Squares	Degrees of Freedom	Mean Square	F-Value	*p*-Value	Comment
Model	0.4627	7	0.0661	47.32	<0.0001	significant
Lack of Fit	0.0046	5	0.0009	0.4571	0.7934	not sign.
Pure Error	0.0080	4	0.0020			
Cor Total	0.4753	16				

**Table 6:** ANOVA for the model for prediction of flexural maximum force $F_{m,f}$.

Source	Sum of Squares	Degrees of Freedom	Mean Square	F-Value	*p*-Value	Comment
Model	1267.96	8	158.50	43.17	0.0001	significant
Lack of Fit	15.35	4	3.84	1.09	0.4661	not sign.
Pure Error	14.02	4	3.51			
Cor Total	1297.33	16				

**Table 7:** ANOVA for the model for prediction of flexural Young’s modulus $E_{f}$.

Source	Sum of Squares	Degrees of Freedom	Mean Square	F-Value	*p*-Value	Comment
Model	0.4944	8	0.0618	22.56	0.0001	significant
Lack of Fit	0.0100	4	0.0025	0.83	0.5678	not sign.
Pure Error	0.0119	4	0.0030			
Cor Total	0.5163	16				

**Table 8:** ANOVA for the model for prediction of compressive maximum force $F_{m,c}$.

Source	Sum of Squares	Degrees of Freedom	Mean Square	F-Value	*p*-Value	Comment
Model	3.590 × 10^7^	9	3.989 × 10^6^	371.55	<0.0001	significant
Lack of Fit	51,266.48	3	17,088.83	2.86	0.1680	not sign.
Pure Error	23,892.16	4	5973.04			
Cor Total	3.598 × 10^7^	16				

**Table 9:** ANOVA for the model for prediction of compressive Young’s modulus $E_{c}$.

Source	Sum of Squares	Degrees of Freedom	Mean Square	F-Value	*p*-Value	Comment
Model	0.1611	8	0.0201	115.28	<0.0001	significant
Lack of Fit	0.0010	4	0.0002	2.47	0.2011	not sign.
Pure Error	0.0004	4	0.0001			
Cor Total	0.1625	16				

**Table 10:** Model’s performance metrics.

Metric	Tensile		Flexural		Compressive	
	$F_{m,t}$	$E_{t}$	$F_{m,f}$	$E_{f}$	$F_{m,c}$	$E_{c}$
$R_{adj}^{2}$	0.9956	0.9530	0.9547	0.9151	0.9952	0.9828
$R_{pred}^{2}$	0.9911	0.9173	0.8449	0.7974	0.9806	0.9029
RMSE	8.95	0.0374	1.92	0.0523	103.62	0.0132
Adeq. precision	70.58	27.30	21.49	16.40	70.38	46.56

**Table 11:** Summarized table of printing parameter influence on mechanical properties.

	Tensile		Flexural		Compressive	
**Printing Parameter**	$F_{m,t}$, N	$E_{t}$, GPa	$F_{m,f}$, N	$E_{f}$, GPa	$F_{m,c}$, N	$E_{c}$, GPa
Layer height	−	−	−	−	−	−
Wall thickness	+	+	+	+	+	+
Infill density	+	+	+	+	+	+
Build plate temperature	+	+	+	+	+	−
Printing speed	−	−	−	−	−	−
Printing temperature	+	+	+	+	+	+

**Table 12:** Confirmation tests with percentage relative errors (coded units).

Specimen No.	Factors						Tensile		Flexural		Compressive	
	$x_{1}$	$x_{2}$	$x_{3}$	$x_{4}$	$x_{5}$	$x_{6}$	$F_{m,t}$, N	$E_{t}$, GPa	$F_{m,f}$, N	$E_{f}$, GPa	$F_{m,c}$, N	$E_{c}$
1	−1	1	−0.333	0.667	0.5	0.333	2.41%	0.75%	2.14%	0.95%	2.73%	10.92%
2	1	−1	0.333	−0.667	−0.5	−0.333	3.33%	0.34%	8.09%	0.06%	4.65%	0.34%
3	0	−1	−0.667	0.333	1	−1	4.50%	2.22%	2.05%	7.47%	6.16%	9.73%
Average							3.41%	1.10%	4.09%	2.83%	4.51%	7.00%

## Data Availability

All data are included in the paper.
